# Waking the Wrong Man

**Published:** 1881-03

**Authors:** 


### WAKING THE WRONG MAN.

The fact that every business has its particular lingo, which is a dead language to people of other professions, was never more clearly shown than in the following article by Bill Nye in the Chicago *Tribune:*

One night, about half after twelve, I judge, I heard somebody step along to the window of my boudoir. Hearing it at that time of night I reckoned that something crooked was going on, so I slid out of bed and got my great blood-searcher and liver purifier, with the new style of centre fire and cartridge ejector, and slid up to the window, calculating to shove a tonic into whoever it might be that was picnicking around my claim.

I looked out so as to get a good idea of where I wanted to sink on him, and then I thought before I mangled him I'd ask him if he had any choice about which part of his vitals he wanted to preserve, so I sung out to him :

"Look out below there, pard, for I'm going to call the meeting to order in a minute. Just throw up your hands, if you please, and make the grand hailing sign of distress, or I'll have to mutilate you! Just show me about where you'd like to have the fatal wound, and be spry about it, too, because I've got my brief costume on, and the evening air is chill!"

He didn't understand me apparently, for a gurgling laugh welled up from below, and the party sings back :

"Hullo, Fatty, is that you? Just looking to see if you'd fired up yet. You know I was to come around and flag you if second seven was out. Well, I've been down to the old man's to see what's on the board. Three is two hours late and four is reported on time. There's two sevens out and two sections of nine. Skinney'll take out first seven and Shorty'll pull her with 102. It's you and me for the second seven, with Limber Jim on the front end, and Frenchy to hold down the caboose. First five is wrong side up in a wash-out this side of Ogallala, and old What's-his-name that runs 358 got his crown sheet caved in and telescoped his headlight into the New Jerusalem. You know the little Swede that used to run extra for Old Hotbox on the emigrant for a while? Well, he's firing 258, and he's under three flats with a coal-oil tank, with a break beam across his coupler and his system more or less relaxed. He's gone to the sweet subsequently, too. Rest of the boys are more or less demoralized and side-tracked for repairs. Now, you don't want to monkey around much, for if you don't loom up like six bits and go out on the track, the ole man 'll give you a time check and the oriental grand bounce. You hear the mellow trill of my bazoo ?"

Then I slowly uncorked the great blood purifier, and moving to the foot-lights where the silvery moonbeams could touch up my dazzling outlines, I said:

"Partner, I am pleased and gratified to have met you. I don't know the first ding busted thing you have said to me; but that's my misfortune. I am a plain miner, and my home is the digestive apparatus of the earth, but for professional melody of the chin you certainly take the cake. You also take the cake-basket and what cold pie there is on the dump. My name is Woodtick William. I discovered the Feverish Hornet up on the Slippery Ellum. I am proud to know you. Keep right on getting more and more familiar with your profession, and by-and-by, when nobody can understand you, you will be promoted and respected, and you will at last be a sleeping-car conductor and revel in the biggest mental calm and wide shoreless sea of intellectual stagnation that the world ever saw. You will—"

But he was gone.

Then I took the pillow-sham and wiped some of the pulverized crackers off the soles of my feet, and went to bed.

